# The Variable Angle Hip Fracture Nail Relative to the Gamma 3: A Finite Element Analysis Illustrating the Same Stiffness and Fatigue Characteristics

**DOI:** 10.1155/2013/143801

**Published:** 2013-03-26

**Authors:** Amir Matityahu, Andrew H. Schmidt, Alan Grantz, Ben Clawson, Meir Marmor, R. Trigg McClellan

**Affiliations:** ^1^Department of Orthopaedic Surgery, UCSF/SFGH Orthopaedic Trauma Institute, 2550 23rd Street, 2nd Floor, San Francisco, CA 94110, USA; ^2^Department of Orthopaedic Surgery, University of Minnesota, Hennapin County Medical Center, Minneapolis, MN 55454, USA; ^3^Anthem Orthopaedics VAN, LLC, Santa Cruz, CA 95060, USA

## Abstract

Ten percent of the 250,000 proximal femur fractures that occur in the United States each year are malreduced into a varus position after treatment. Currently, there is no cephalomedullary nail available that allows the physician to dynamically change the lag-screw-to-nail angle. The Variable Angle Nail (VAN) was designed to allow movement of the lag screw relative to the shaft of the nail. This study compared the characteristics of the VAN to the Gamma 3 nail via finite element analysis (FEA) in stiffness and fatigue. The results of the FEA model with the same loading parameters showed the Gamma 3 and the VAN with lag-screw-to-nail angle of 120° to have essentially the same stiffness values ranging from 350 to 382 N/mm. The VAN with lag-screw-to-nail angles of 120°, 130°, and 140° should be able to withstand more than 1,000,000 cycles from 1,400 N to 1,500 N loading of the tip of the lag screw. The Gamma 3 should be able to last more than 1,000,000 cycles at 1,400 N. In summary, the VAN is superior or equivalent in stiffness and fatigue when compared to the Gamma 3 using FEA.

## 1. Introduction

More than 250,000 proximal femur fractures occur in the United States each year [1]. Due to aging of the population and the increasing number of elderly persons, the incidence of hip fractures is predicted to double over the next 15 to 20 years [1]. Since their introduction over 15 years ago and subsequent widespread adoption, the so-called cephalomedullary nails that incorporate fixation into the femoral head have become a standard treatment for these proximal femur fractures [2–7]. Despite their popularity, the technology associated with these nails has only been incrementally advanced over the last 10–15 years, with most of the design changes having been done to lessen the likelihood of intraoperative fracture of the femur and cutout of the lag screw [8, 9] Reports of the results of the stabilization of proximal femoral fractures with cephalomedullary nails indicate that more than 10% of cases demonstrate varus malreduction, with neck-shaft angles that are more than 10° of varus relative to the contralateral side [10, 11]. Varus malreduction contributes to failure of fracture fixation and causes limb shortening and compromised function [12, 13]. Given the number of patients treated by this method, as many as 20,000 individuals per year may have a suboptimal result due to malreduction, and some of these patients may require further surgery [1, 7, 10, 11]. It is recognized that patient outcomes, fracture nonunion, and implant failure are largely dependent on the fracture reduction and less on which specific intramedullary implant is used [14–17]. The etiology of functional impairment following the surgical treatment of intertrochanteric hip fractures is multifactorial. However altered biomechanics are likely to be a contributing factor to poor functional outcomes. Healing with shortening or varus malreduction alters the abductor lever moment arm and femoral head offset. Compromised abductor strength may result in a residual deficit in gait and can have a significant impact on the functional outcome and may require corrective surgery [18]. Satisfactory functional outcomes with near normal gait restoration have been demonstrated in patients with intertrochanteric hip fractures treated with a trochanteric entry nail and accurate anatomic reduction [19–28].

Unfortunately, despite incremental design advances over the last 15 years, surgeons still do not have a means of correcting intraoperative varus malreduction in situ when it occurs. Current implant designs incorporate a static lag-screw-to-nail angle curtailing the ability of the surgeon to change the reduction of the fracture once the implant is placed. The Variable Angle Nail (Anthem Orthopaedics VAN, LLC, Los Altos, CA) has been designed as a solution for the problem of intraoperative proximal femur varus neck-shaft angle malreduction. Because the lag-screw-to-nail angle can be changed intraoperatively to correct malreductions, one can choose the correct angle for the patient's anatomy. However, because the mechanism within the nail is unique, with multiple parts, and is dynamic rather than static, there is need to demonstrate the ability of the implant to withstand expected mechanical loads during clinical use of slightly more than 2 body weights [29–32]. Moreover, because there is a mobile internal apparatus, load transfer may be different between the lag screw, internal apparatus, and nail relative to current static designs. Additionally, the site of internal material stress and potential failure of the novel implant may be different relative to current designs. In current designs of cephalomedullary nails there are compressive forces laterally and medially that cause shear stress at the anterior and posterior aspect of the nail [33]. However, because of the nature of the internal mechanism of the VAN, there may be differences in force transfer from the lag screw to the nail.

The purpose of this finite element analysis study was to describe the areas of high stress and location of failure in the VAN as compared to the Gamma 3 (Stryker, Kalamazoo, MI). Of special interest were areas of high stress concentration at the interface between the lag screw and the nail aperture and internal mechanisms for both the VAN and Gamma 3. 

## 2. Methods

Finite element analysis (FEA) of the VAN and Gamma 3 intramedullary nails was performed in several steps. Modeling the implants, testing parameters, material characteristics, static calculations, and fatigue calculations were performed as described in the following.

### 2.1. VAN Description

**Figure 1:**
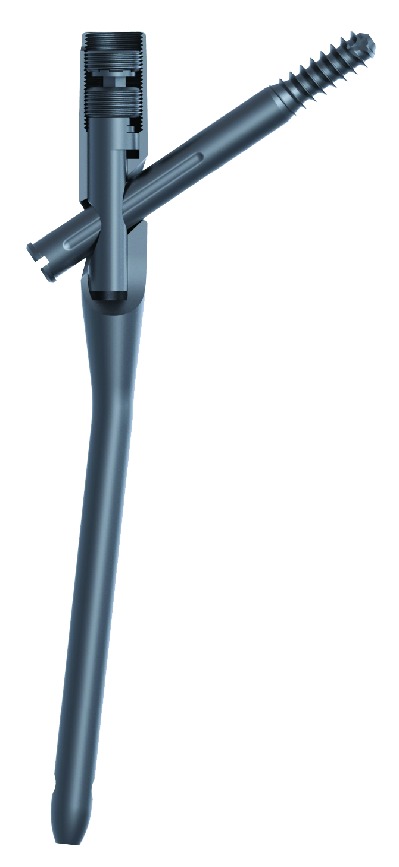
The Variable Angle Nail (VAN) with internal mechanism that gives the surgeon the opportunity to move the lag screw relative to the nail.

The Variable Angle Nail (VAN) is comprised of a nail, a lag screw, and an internal mechanism that dynamically moves the lag screw from 120° to 140° of lag-screw-to-nail angles (Figure 1). The surgeon may be able to intraoperatively and in situ move the lag screw to a desired position, thereby accommodating any patient-specific anatomic variations. Unlike the Gamma 3, which has a proximal lateral bend of 4°, the proximal aspect of the VAN is bent 6° laterally. Both proximal nails accommodate for lateral entry point and the proximal femoral anatomy.

### 2.2. Modeling

The FEA model for the VAN and Gamma 3 was built from a SolidWorks computer-aided design assembly (SolidWorks Corporation, Concord, Massachusetts). Autodesk Simulation software was used to build and analyze the model (Autodesk, Inc., San Rafael, CA). The model consists of approximately 165,000 elements. A hybrid-meshing scheme was used in which 8-node brick elements comprise the majority of the volume. Fewer 6-node wedge, 5-node pyramid, and 4-node tetrahedral elements were used to close voids where needed. The material model accounted for plasticity in the nail, lag screw, and insert. The nail, lag screw, and insert were the only parts where yield occurred as a result of contact stresses. Surface-to-surface contact elements were employed between lag screw and nail, lag screw and insert, insert and nail, and insert and adjustment screw. The surface-to-surface contact was designed so that the nodes of either surface were prevented from passing through the nodes defining the other surface. A contact tolerance of 0.02 mm was used to define the gap value below which contact is assumed to occur. Frictionless contact was used, as vibration during cyclic testing was assumed to overcome frictional restraint between the parts. An adaptive contact stiffness algorithm was used by the FEA program to calculate contact stiffness. A nonlinear iterative solution method (Newton-Raphson) was used with line search Scheme. The Newton-Raphson method generates a search direction for new possible solutions, while the line search scheme is used to find a solution in the direction that minimizes the out-of-balance force error. The convergence criterion was displacement since the last time step. The convergence tolerance was Δ*d*/*D* < 0.005 with contact and <0.0001 without contact.

Three versions of this first model set were built with aperture angles of 120°, 130°, and 140° between the lag screw and the nail.

A separate, second model of the interaction between the insert, adjustment screw, and nail was built and analyzed as well to delineate any high stress areas that may be transferred proximally from the lag screw to the insert and then to the adjustment screw and its threads that interact with the nail. This second one, or submodel, was created for the VAN design so that the stress levels in the threads and other features of the adjustment screw and its interface to the nail could be accurately modeled. Accurately modeling the contact elements in the threads of the adjustment screw to nail interface would have resulted in an unacceptably large model. In the full VAN model, contact elements are used between all the parts except the adjustment screw to nail interface and the nail to potting compound (both considered bonded). In the VAN model there were 12 sets of mating surfaces with contact elements.

The adjustment screw was considered bonded to the nail in the first model of the VAN in order to focus on the interactions of the lag screw and the insert laterally and the lag screw and the nail medially. A third model (two versions) was created for the Gamma 3 nail with the aperture angles of 120° and 130° angles between the lag screw and the nail.

### 2.3. Set-Up

**Figure 2:**
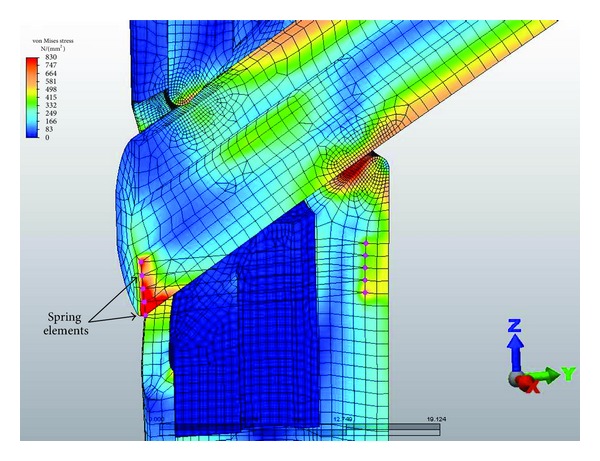
FEA model of the interaction between the insert, adjustment screw, and nail showing the spring elements that control collapse of the lag screw within the aperture of the nail.

**Figure 3:**
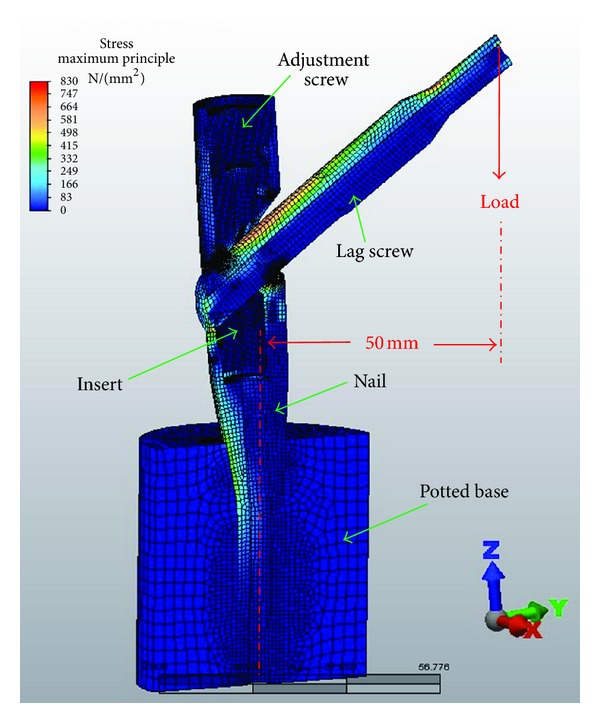
FEA model including loading set-up of 50 mm offset from the nail to the point of force application.

A potted base was modeled to simulate the plastic mount for the nail used during in vitro testing. A hose clamp was emulated with multiple springs placed between the end of the truncated lag screw and the medial side of the nail distal to the lag screw aperture in order to constrain the lag screw from sliding laterally. Five springs were used with a stiffness of 1000 N/mm for each spring (Figure 2). The parts and loads were symmetric about the *Y*-*Z* plane. The load was offset 50 mm from the lower axis of the nail (Figure 3). 

### 2.4. Material Characteristics

The VAN and Gamma 3 are both made from a titanium alloy (Ti-6Al-4V ELI). The stress-strain curve used in the FEA model was derived from a curve for annealed Ti-6Al-4V found in the Atlas of Stress-Strain Curves [34]. The curve is modified to account for the yield stress of the ELI alloy of 790–795 MPa and the necking during stressing that occurs during loading. It is also important to note that the following values were taken into account in regard to material properties of Ti-6Al-4V ELI (Table 1).

Since the areas of high contact in the Gamma 3 and VAN nail are bearing type stresses, the local yield stresses may be exceeded without causing component failure.

### 2.5. Static Loading and Stiffness Calculations

The Gamma 3 was tested at the worst-case scenario of 120° lag-screw-to-nail angle with a load of 1400 N directed distally on the lag screw. We chose the 120° because the resultant force vector loading the nail is more horizontal. Therefore, there is an increase in lever arm affecting the nail with an increased lag-screw-to-nail angle. The VAN was tested at 120°, with 1400 N load, and then at 120°, 130°, and 140° lag-screw-to-nail angles at 1500 N. We chose 1500 N because it is slightly more than two times average body weight. 

The stiffness plots for the VAN system were calculated using the 50 mm offset from the tip of the lag screw to the shaft of the nail. By necessity, the length of the lag screws is increased in length as the lag-screw-to-nail angle increased from 120° to 140°. The nail proximal bend angle of the Gamma 3 is 4° versus 6° for the VAN system resulting in a shorter lag screw moment arm length for the Gamma 3 (61 mm) versus the VAN (64 mm). The distance from the intersection of the lag screw axis from the center of the nail to the center of the load applicator was 64 mm, 72 mm, and 86 mm, respectively, for 120°, 130°, and 140° lag-screw-to-nail angles. For purposes of comparison, the stiffness values for both the VAN and Gamma 3 with 64 mm moment arms were used. The predicted stiffness for the Gamma 3 with a 64 mm moment arm is 350 N/mm between 0 and 1500 N.

The measured test results for the stiffness of the VAN system from 0 to 1500 N were 340 N/mm for a 120° lag screw angle and an average value of approximately 310 N/mm for 130° and 140° lag screw angles. 

### 2.6. Fatigue Calculations

The level of static tensile stress that should not be exceeded if 1,000,000 load cycles were to be achieved before failure was chosen to evaluate the fatigue capability of the VAN and Gamma 3 designs (see Appendix A).

The data for Ti-6AL-4V lists a fatigue strength of 74,000 psi (510 MPa) at 10,000,000 cycles, and the ultimate strength is 131,000 psi (900 MPa) [35, 36]. Because an average person walks 1 million steps in 3 months, 1,000,000 cycles were chosen as the fatigue longevity of the implants for this FEA [37]. Using the stress range formula (Appendix B), the maximum allowable range of stress for 1,000,000 cycles is 566 MPa and for 200,000 cycles it is 605 MPa. 

The potential for compressive fatigue was further evaluated by comparing the von Mises stresses with the maximum and minimum principal stresses in the regions of high compressive loading in the nail and insert. The von Mises stress is most commonly used in noncyclic FEA as the best prediction of the material yield point in a multiaxial stress state. The von Mises calculation utilizes distortion energy to find an equivalent value of uniaxial stress, which can be compared to yield point test data. In fatigue analysis, the failure limits in tension and compression can be quite different. Loading in compression can be significantly higher than loading in tension to achieve the same fatigue lifetime.  Figure 8 illustrates that the tensile component (maximum principal stress) is much lower than the magnitude of the compressive component (minimum principal stress). The magnitude of the minimum principal stress is approximately equal to the von Mises stress. This shows that the stresses in these regions are predominantly compressive. Using the maximum principal stress component as the basis of the fatigue life calculation explains the test results showing that the VAN system can reach a 1,000,000-cycle fatigue life under high loads. At the actual regions of contact, some plastic deformation occurs, and the loads are redistributed. These contact regions are seen in the bearing stress illustration in Figure 7.

 The stress state in the VAN and Gamma 3 analysis are comprised of an average stress and a range stress. In the Soderberg diagram (Appendix C), the average stress is 0.55 Smax, and the range stress is 0.45 Smax. 

Therefore, for a fatigue life of 1,000,000 cycles, Se = 566 MPa, and solving for Smax, the maximum tensile stress of the FEA plots should be less than 686 MPa. Moreover, for a fatigue life of 200,000 cycles, the maximum stress on the FEA plots should be less than 711 MPa. In other words, a peak tensile stress of 686 Mpa should not be exceeded at the maximum principal stress areas located on the medial and lateral parts of the nail and nail-insert. This location, the aperture for the lag screw in the Gamma nail, has been found to be the most common location of nail breakage [38, 39]. The highest stresses occur in the contact regions between the lag screw and nail and the lag screw and insert. These regions are in compression. The compressive yield strength of this Ti alloy is 860 MPa, while the ultimate bearing strength is 1740 MPa. Testing has shown that these regions are not prone to fatigue failure.

## 3. Results

**Figure 4:**
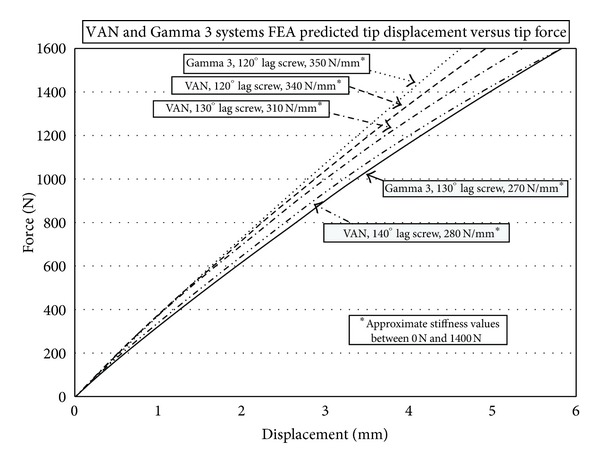
Force displacement curve of the VAN at 120°, 130°, and 140° and the Gamma 3 at 120° and 130° lag-screw-to-nail angles. The initial stiffness values were VAN 120°, 340 N/mm; VAN 130°, 310 N/mm; VAN 140°, 280 N/mm; Gamma 3 120°, 350 N/mm; Gamma 3 130°; 270 N/mm.

The FEA model illustrated that the 120° Gamma 3 and the 120° VAN had essentially the same stiffness values of 350 N/mm and 340 N/mm, respectively (Figure 4). However; the 130° and 140° VAN was stiffer than the 130° Gamma 3. 

**Figure 5:**
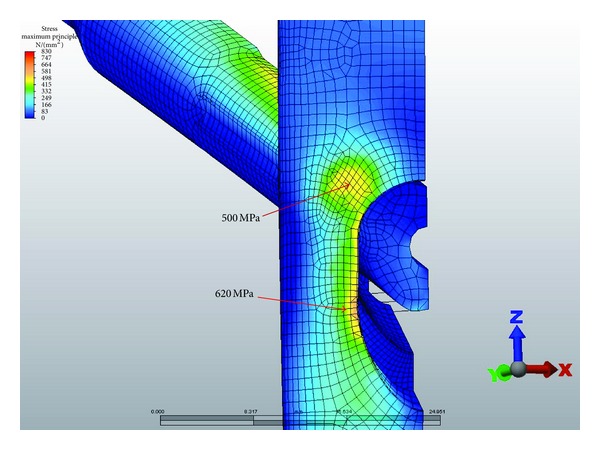
120° lag-screw-to-nail angle VAN stresses. The maximum stress was 500 MPa at the proximal aspects and 620 MPa at the distal aspect of the lateral aperture.

**Figure 6:**
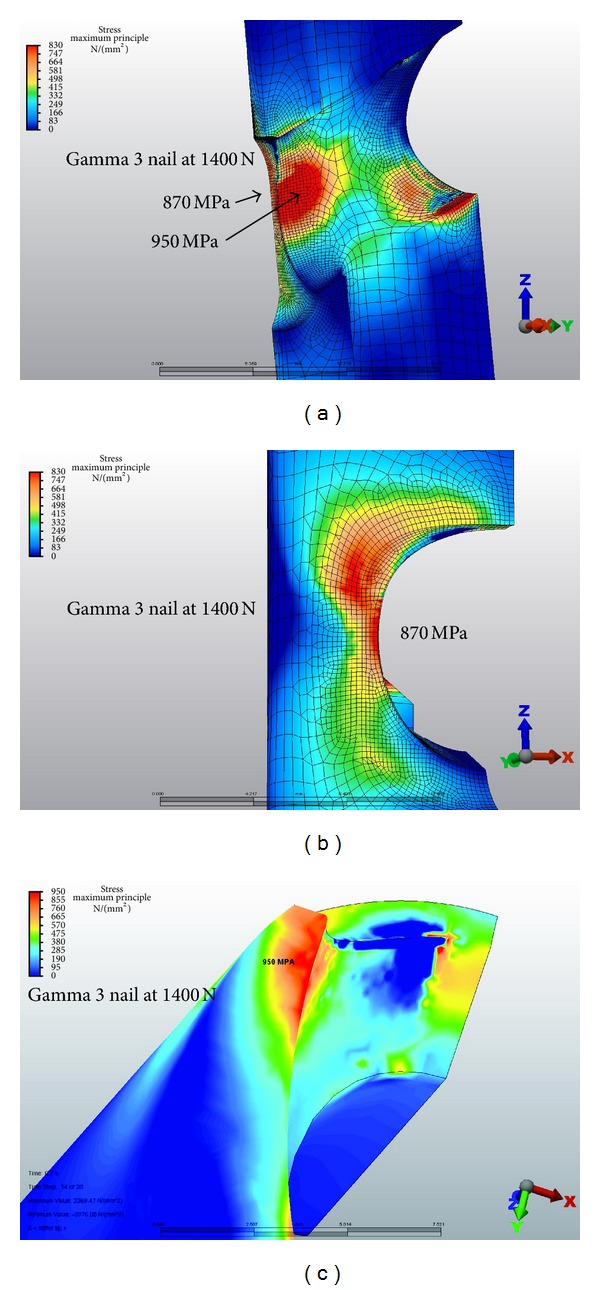
120° lag-screw-to-nail angle Gamma 3 stresses. The maximum stress was 870–950 MPa at the lateral and medial aspect of the nail.

**Figure 7:**
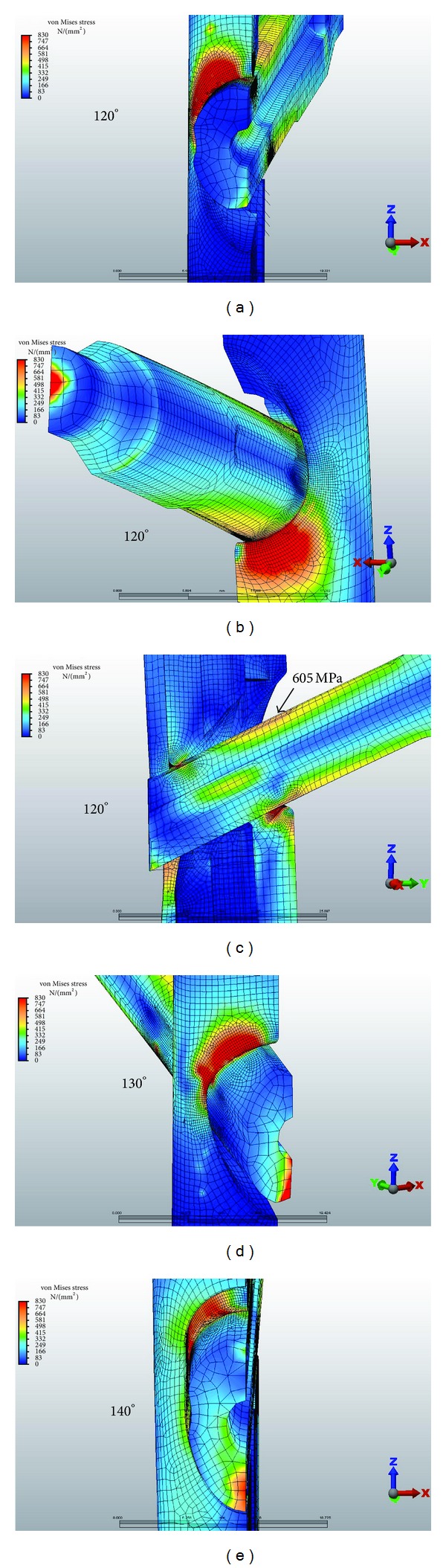
1500 N of force on the lag screw with subsequent stresses on the VAN at 120°, 130°, and 140° lag-screw-to-nail angles.

**Figure 8:**
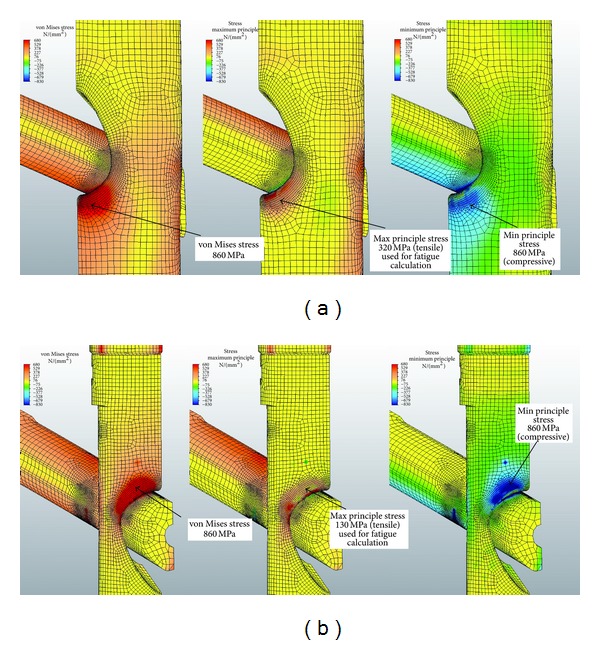
1500 N of force on the lag screw with subsequent von Mises and principal stresses compared at 120° lag screw-to-nail angle.

**Figure 9:**
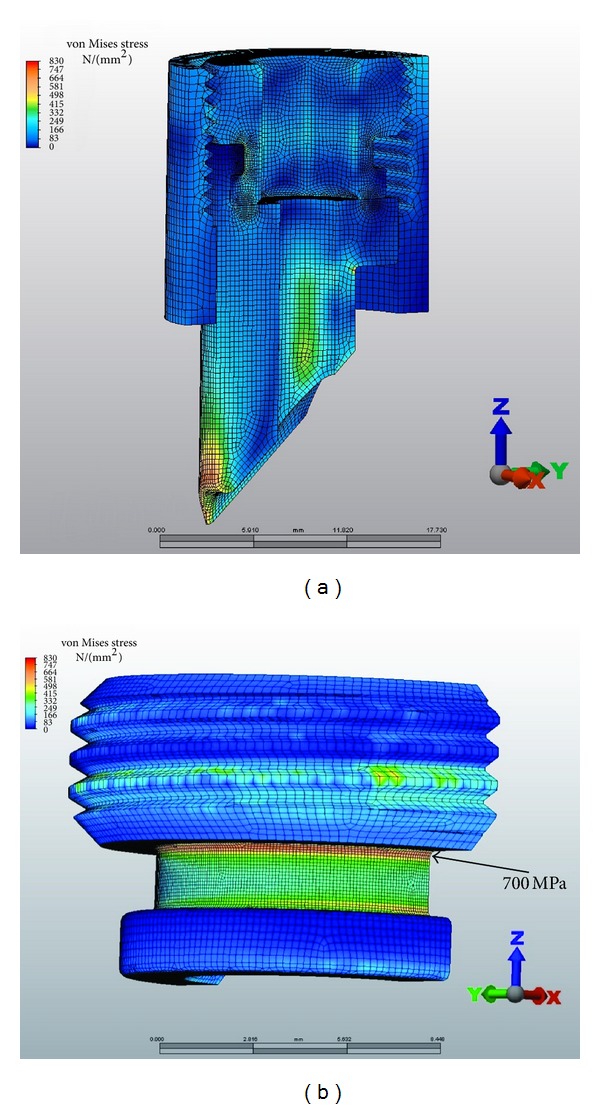
Adjustment screw interactions with insert stresses at 1500 N.

 Based on the fatigue formula calculations described previously, the peak tensile stress derived by the FEA should not exceed 680 Mpa. At 1400 N of loading, with the VAN set to 120° lag-screw-to-nail angles, the maximum stress was 500 MPa at the proximal aspect and 620 MPa at the distal aspect of the lateral aperture (Figure 5). A Gamma 3 nail with a similar lag-screw-to-nail angle had a maximum stress of 870–950 MPa at the lateral and medial aspect of the nail (Figure 6). At 1500 N of force on the lag screw, subsequent tensile stresses on the VAN at 120°, 130°, and 140° lag-screw-to-nail angles were on average less than 680 Mpa, although local areas of stress approaching the fatigue limit were observed (Figure 7).  Figure 8 illustrates that the tensile component (maximum principal stress) is much lower than the magnitude of the compressive component (minimum principal stress). The magnitude of the minimum principal stress is approximately equal to the von Mises stress. This shows that the stresses in these regions are predominantly compressive. Using the maximum principal stress component as the basis of the fatigue life calculation explains the test results showing that the VAN system can reach a 1,000,000-cycle fatigue life under high loads. At the actual regions of contact, some plastic deformation occurs, and the loads are redistributed. These contact regions are seen in the bearing stress illustration in Figure 7. The FEA calculated stresses in the adjustment screw were also below 680 Mpa (Figure 9). 

## 4. Discussion

This study was performed to evaluate the mechanical properties of the VAN relative to the Gamma 3. Finite element analysis was performed in order to evaluate the stresses that are transferred from the lag screw onto the VAN during normal loading conditions as compared to the Gamma 3 nail. The analysis suggests that the VAN internal mechanism and nail have a decrease in stresses when compared to the Gamma 3 during nearly similar loading conditions (1400 N and 1500 N). 

Typically, Ti alloy nail implants placed in the proximal femur to fix hip fractures fail in tension at the distal aspect of the lateral lag screw aperture (Figure 5). The allowable maximum principal stress for fatigue testing was calculated to be less than 680 MPa. The FEA demonstrated maximal principal tensile stress of up to 620 MPa in the VAN at this location. Calculations of fatigue strength and compression stress indicate that the VAN should be capable of withstanding 1,000,000 cycles at 1400 N at 120°, 130°, and 140°. At 1500 N force on the lag screw, the VAN at 120°, 130°, and 140° neck-shaft angles should pass 1,000,000 cycles, although the lag screw in the 130° setting was approaching the maximum allowable stress. In comparison, the Gamma 3 system is capable of 1,000,000 cycles at 1400 N at 120°. 

Given the expected increasing number of proximal femur fractures, variety of anatomic variations in neck-shaft angles and the associated prevalence of varus malreduction following cephalomedullary nailing [1, 10, 11], the VAN may offer a simple intraoperative solution to solve some of these problems. 

The limitations of this study may be due to implant modeling, testing set-up assumptions, or computational testing analysis. When designing this study, we sought to minimize any implant modeling error by developing virtual nails from actual implants. The high mesh density of the FEA models resulted in close approximations of the actual nail systems studied. The models consist of approximately 165,000 solid elements, the majority of which are 8-node bricks. In a few regions where 8-node bricks would result in an unacceptably high mesh density, 6-node wedge, 5-node pyramid, and 4-node tetrahedral elements are used to close voids in the model. Material properties were accounted for by allowing for plasticity in the nail, lag screw, and insert. 

In clinical practice, the lag screw tends to slide within the nail aperture in patients that are treated with these types of implants. As the lag screw slides, the lever arm decreases and minimizes the stresses seen within the nail. The worst-case situation was emulated by holding the lag screw to the nail by spring elements that did not allow the lag screw to slide. This could change the mechanical environment slightly. However, since the nails were matched to each other, this effect was probably minimized in the comparison between them. By following protocols commensurate with previous studies that evaluated the stiffness and fatigue of implants of this nature, the testing set-up error was minimized. Moreover, established mechanical and FEA testing parameters that exist for proximal femoral fracture nails were employed in order to create uniform testing [33].

One limitation of this study is that there is no previously published mechanical testing of the VAN system. Moreover, there is no in vivo analysis of strain, or even FEA, of the Gamma 3 nail in the unstable hip fracture model that we are aware of. Therefore, although the authors tried to test these two implants relative to each other, the applicability to clinical use is not established as of yet.

In summary, the finite element analysis comparing the Variable Angle Nail (VAN) to the Gamma 3 nail illustrated that there are minimal differences in stiffness or fatigue between the two nails but with the potential advantage changing dynamically the angle between the lag screw and accommodating multiple patient multiple femoral neck-shaft angles with a single nail. Future mechanical testing of strength and fatigue should be performed to further evaluate the feasibility of using the VAN in clinical practice. 

## Figures and Tables

**Figure 10:**
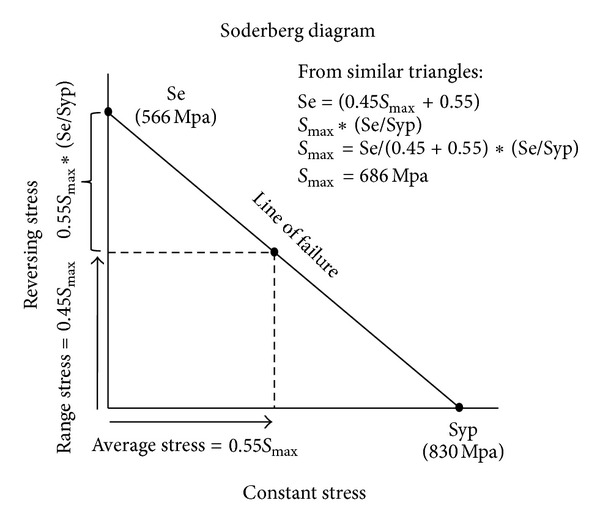
Soderberg diagram.

**Table 1:** 

Material properties of Ti-6Al-4V ELI	Strength in MPa
Ultimate tensile strength	860
Tensile yield strength	790
Compressive yield strength	860
Ultimate bearing strength	1,740
Bearing yield strength	1,430

## Appendices

### A. Fatigue Range and Total Stress Calculations

Published values of fatigue strength are based on fully reversing induced stresses. In the case of the VAN System, the testing protocol loads the lag screw from 10% to 100% of the applied load. This results in minimum and average stresses of 10% maximum stress and 55% maximum stress respectively. The average stress is (10% of maximum + 100% of maximum)/2 = 55% of maximum. The range, or fluctuating stress is (100% of maximum − 10% of maximum)/2 = + 45% of maximum. The range stress is applied as a sine wave, so the range stress = 45%  max stress ∗ sin (wt). The total excursion of the range stress is 90% of the maximum stress. Since the published values of fatigue strength are based on fully reversing stresses, a corresponding maximum allowable stress value must be determined. This maximum allowable stress is then compared to the stress levels predicted by the FEA in order to determine a margin of safety.

Therefore, the total stress = 55% max + 45% max ∗ sin (wt).

### B. Fatigue Strength Calculations

Using a semi-log plot, the fatigue strength, Sr at 1E6 cycles is: Sr = Sult − ((Sult − Se)/7)log⁡10(N), Sr = 131,000 − ((131,000 − 74,000)/7)log⁡10(10^6^) = 82,100 psi (566 MPa).

Sr: stress range; Sult: ultimate stress; Se: Se-fatigue endurance stress at 1E7 cycles.

Se is the allowable value of fully reversing range stress, Sr.

Since the stress state is not fully reversing, but instead varies from 10% of maximum stress to 100% of maximum stress, we determine the maximum allowable stress, Smax, which results in the same fatigue life (1E6 cycles) as the fully reversing stress, Sr.

### C. Soderberg Diagram

*S *_max_ is calculated to be 686 MPa (see Figure 10).
